# Transformation and fate of Fe(III) in petroleum-hydrocarbon-contaminated soil and groundwater

**DOI:** 10.1186/s12932-025-00097-z

**Published:** 2025-02-07

**Authors:** Essouassi Elikem, David Bulmer, Kris Bradshaw, Ardalan Hayatifar, Matthew B. J. Lindsay, Steven D. Siciliano, Derek Peak

**Affiliations:** 1 Department of Chemistry, University of Saskatchewan, 170 Thorvaldson Building, Saskatoon, S7N 5E2 SK Canada; 2 Department of Soil Science, University of Saskatchewan, 51 Campus Drive, Saskatoon, S7N 5A8 SK Canada; 3 Federated Cooperatives Ltd., 401 22nd Stret East, Saskatoon, S7H 0H2 SK Canada; 4 Department of Geological Sciences, University of Saskatchewan, 114 Science Place, Saskatoon, S7N 5E2 SK Canada

**Keywords:** *In situ* bioremediation, Petroleum hydrocarbons, Fe(III) transformation, XANES spectroscopy

## Abstract

In anoxic subsurface environments, low Fe(III) bioaccessibility greatly limits *in situ* biodegradation of petroleum hydrocarbons (PHCs). Ferric ammonium citrate is a soluble compound that has the potential to increase the bioaccessibility of Fe(III). However, in neutral to alkaline environments, Fe(III) hydrolysis can produce Fe(III) (oxyhydr)oxides that may subsequently transform or recrystallize to relatively stable and less bioaccessible phases. Accordingly, the objective of this study was to elucidate the transformation and fate of Fe(III) contributed by ferric ammonium citrate in a gasoline-contaminated subsurface environment that was undergoing *in situ* bioremediation. Ferric ammonium citrate, together with sodium tripolyphosphate, magnesium sulphate, and nitric acid, was continuously injected into the contaminated groundwater for about 22 weeks. Colloids in the groundwater (solid particles retained on a 0.45 $\upmu$m filter) and soil cores were collected from the site. Fe speciation in these samples was characterized using X-ray absorption near edge structure (XANES) and Fourier transform infrared (FTIR) spectroscopy. The groundwater colloids (GWCs) contained mostly octahedrally coordinated Fe(III), but the subsoils contained both octahedrally coordinated Fe(III) and Fe(II). The fraction of Fe(II) in the subsoils generally increased after about 22 weeks of continuous amendment injection. Ferric ammonium citrate did not persist in the PHC-contaminated subsurface: the Fe(III) it contained was transformed to solid phases. Fe(III)-organic-matter (Fe(III)-OM) complex/coprecipitate and sulfate green rust were the major phases present in the GWCs; akaganeite, chloride green rust, vivianite, ferrihydrite, Fe(III)-silicate, and magnetite were present as minor phases. The subsoils contained three major phases: Fe(III)-OM complex/coprecipitate, magnetite, and calcium ferric silicate. The presence of major Fe(II) phases in the subsoils strongly indicate that secondary Fe(III) phases (especially Fe(III)-OM complex/coprecipitate) served as terminal electron acceptors during the microbial degradation of PHCs in the contaminated subsurface.

## Introduction

In anoxic subsurface environments in which Fe(III) is the terminal electron acceptor, microbes degrade petroleum hydrocarbons (PHCs) if poorly crystalline Fe(III) (oxyhydr)oxides and essential nutrients (e.g., N and P) are not limiting. However, when poorly crystalline Fe(III) (oxyhydr)oxides are unavailable, the transport and biogeochemical processes that regulate their availability control microbial degradation of PHCs [4, 47]. Under natural subsurface conditions, the rate at which transport and biogeochemical processes replenish amorphous Fe(III) (oxyhydr)oxides is slow; hence the persistence of petroleum hydrocarbons in subsurface environments.

Previous laboratory studies have shown that chelating agents (e.g., citrate) and chelated Fe(III) species (e.g., ferric citrate) can increase the availability of Fe(III) [25, 26]. For this reason, at hydrocarbon-contaminated sites having low levels of Fe(III), soluble ferric compounds are injected into the subsurface [4, 9, 38]. However, it is uncertain whether a relatively high soluble Fe(III) concentration can be maintained in soils and groundwater after the addition of chelating agents or chelated Fe(III) species. Injecting these soluble compounds into the subsurface may initially increase aqueous Fe(III) concentration. At pH above 7, however, citrate becomes less effective in chelating ferric iron [10]. Consequently, Fe(III) may precipitate as Fe(III) (oxyhydr)oxide colloids and as coatings on mineral and organic substances [3, 19]. The precipitation and subsequent crystallization of Fe(III) (oxhydr)oxides may decrease aqueous Fe(III) concentration, leading to a decrease in Fe(III) availability and reducibility. In addition, Fe(II)-catalyzed dissolution and reprecipitation may transform poorly ordered Fe(III) phases into thermodynamically stable phases [17, 42]. Ferric iron can form soluble and insoluble complexes with organic molecules derived from soil biota [12, 19, 36]. These complexes are the product of the reaction between Fe$^{3+}$ and carboxyl, phenolic, and carbonyl functional groups [7, 43]. The association between Fe(III) and organic molecules may preserve poorly ordered Fe(III) (oxhydr)oxides by preventing Fe(II)-catalyzed transformation [6, 42].

Considering its importance in *in situ* bioremediation of PHCs, the chemical transformation and fate of Fe(III) in groundwater and subsoil will greatly impact the biodegradation of hydrocarbon contaminants. Accordingly, this study sought to determine the fate of Fe(III) contained in ferric ammonium citrate, a chelated Fe(III) compound that was injected into the PHC-contaminated groundwater and subsoil at a former gas station in Stony Plain, AB, Canada. The overall objective of this paper was to characterize the chemical properties of Fe in the groundwater colloids and subsoils collected from the remedial site.

## Methods

### Site history and site description

From 2011 to 2014, groundwater monitoring projects detected light nonaqueous phase liquid (LNAPL) at the remedial site in Stony Plain, AB, Canada. A dual-phase recovery system was used to remove LNAPL, but some still remained in monitoring wells. In 2013, during the field season, a skimmer unit was used to remove about 48 L of LNAPL from one of the monitoring wells. A Phase II Environmental Site Assessment was conducted in July 2017. Fourteen boreholes were excavated and converted into groundwater monitoring wells. Both borehole soil and groundwater samples were analyzed for PHCs: of the 14 soil and groundwater samples, 10 and 13, respectively, contained PHCs above the regulatory limit. In addition, four monitoring wells contained LNAPL of thickness that ranged between 10 to 55 cm. These results were used to delineate the PHC plumes in the soil and groundwater; they also informed the design and implementation of the *in situ* bioremediation program that was implemented from 2018 to 2019.

The *in situ* bioremediation system consisted of an injection gallery and an amendment solution system. The injection gallery was $\sim$30 m long and $\sim$6 m wide and had three rows of 12 drive-point wells, which were installed 2 m apart. In each row, the depth of the drive point wells alternated between 4 m and 7 m. The injection gallery was connected to the amendment solution system using a 25-mm Tigerflex tubing. A schematic of the injection gallery is provided in Additional file 1: Figure S1. The amendment solution system comprised four 1000-L totes housed in a steel storage container. In 2018, the totes separately contained ferric ammonium citrate $[{(\text{NH}_4)_{5}\text{Fe}(\text{C}_{6}\text{H}_{4}\text{O}_{7})_{2}}]$, sodium tripolyphosphate (${\text{Na}_{5}\text{P}_{3}\text{O}_{10}}$), ${\text{MgSO}_{4} \cdot 7 \text{H}_{2}\text{O}}$ and ${\text{HNO}_{3}}$, and sodium fluorescein (replaced with bone-meal hydrochar in 2019). The totes were metered into a tap water source, and, from May 3 to October 3, 2018, and from May 14 to October 9, 2019, the amendment solutions were injected via peristaltic pumps to the drive-point wells. The concentrations and volumes of each amendment injected are listed in Table 1.

**Table 1:** Concentrations and volumes of amendement solutions injected into the ground water

Tote	Amendement	Concentration	Volume injected (L)	
			2018	2019
1	${(\text{NH}_{4})_{5}\text{Fe}(\text{C}_{6}\text{H}_{4}\text{O}_{7})_{2}}$	2.5 x 10$^{-4}$ mol L$^{-1}$; (13 mg L$^{-1}$ Fe(III))	1431	1412
2	${\text{Na}_{5}\text{P}_{3}\text{O}_{10}}$	1 x 10$^{-4}$ mol L$^{-1}$; (3.1 mg L$^{-1}$ P)	1383	1304
3	${\text{MgSO}_{4} \cdot 7 \text{H}_{20}}$	5.2 x 10$^{-3}$ mol L$^{-1}$; (500 mg L$^{-1}$ ${{\text{SO}^{2-}_{4}}}$)	1438	1625
	${\text{HNO}_{3}}$	2.4 x 10$^{-4}$ mol L$^{-1}$; (3.4 mg L$^{-1}$ N)		
4	Sodium flourescein	2.5 x 10$^{3}$ mg L$^{-1}$	794	−
	Bone-meal hydrochar	10 $\%$	−	400

### Groundwater chemistry and sampling of groundwater colloids

In 2019, groundwater and colloidal (solid particles retained on a 0.45 $\upmu$m filter) samples were collected on two occasions: before (week zero) and after 22.5 weeks of continuous injection of the amendment solutions. On each occasion, groundwater from five monitoring wells was purged at low flow rates (0.1$-$0.5 L min$^{-1}$) and filtered using 0.45-$\upmu$m polyethersulphone filters (Waterra Pumps Limited). The filters were immediately frozen in liquid nitrogen and stored in coolers containing dry ice before they were transferred to –80 $^{\circ }$C freezers. From the same monitoring wells, unfiltered low-flow groundwater samples were collected for chemical analysis. Total organic carbon was measured using the high-temperature combustion method; total and dissolved Fe and Mn, Ca$^{2+}$, Mg$^{2+}$, Na$^{+}$, and K$^{+}$ were measured using inductively coupled plasma mass spectroscopy (ICP-MS); nitrate, nitrite, and sulfate were measured using ion chromatography; chloride and sulfide were measured using, respectively, the automated ferricyanide method and the gas dialysis, automated methylene blue method; total phosphorus was measured using the automated ascorbic acid method. Total alkalinity and pH were determined using the titration method and electrometric method, respectively. Electrical conductivity was measured using a conductivity meter and probe. In each well, redox potential (Eh) and temperature were measured using an Aqua TROLL^®^ 400 multiparameter probe (In-Situ Inc.). The measured parameters are presented in Table 2.

### Sampling of soil cores

Soil cores were collected on two occasions: before (week zero) and after 22.5 weeks of continuous injection of the amendment solutions. The cores were collected using a truck mounted 7822DT Geoprobe rig equipped with 95 mm probe rods; the probe rods were driven into the subsurface with a GH70 percussion hammer. Because the water table at the site was about 3 m, the soil cores were collected from three to six meters below ground level. Each core was sliced into 0.75 m subsections. The subsections were waxed and capped at both ends, kept on ice, and later stored at –20 $^{\circ }$C until further processing.

### Incremental sampling of soil cores

An incremental sampling methodology (ISM) [18] was employed to collect subsamples from each core for Fe speciation analysis. The frozen cores were partially thawed prior to sampling, and the PVC pipe housing was removed with a core cutter. Afterwards, the surface of the cores was scraped off using a putty knife. Using a plastic Terra Core™ sampler (En Novative Technologies, Dexter, USA), about 2 g of soil was collected at 15-cm intervals into empty glass vials. Altogether 20 subsamples were collected from each three-meter core. The subsamples were collected under ambient conditions; therefore, to minimize the oxidation of Fe(II) to Fe(III), the samples were quickly taken from the partially thawed cores and immediately stored at -20 $^{\circ }$C. The samples were later freeze-dried, and the concentration of Fe present was determined using a Bruker S2 Puma X-ray fluorescence spectrometer.

### Fe K-edge X-ray absorption spectroscopy

X-ray absorption spectroscopy (XAS) analysis was performed on the groundwater colloids (GWCs) and soil samples at the IDEAS and HXMA beamlines, respectively, at the Canadian Light Source (CLS) in Saskatoon, SK, Canada. The CLS storage ring operated at 2.9 GeV and the beam current ranged between 150–250 mA. The IDEAS beamline used a Ge(220) double-crystal monochromator with about 0.5 eV resolution. The monochromator was detuned by 50% to reduce higher harmonics and calibrated to the first inflection point of the K-edge (7112 eV) of an Fe reference foil. Before the X-ray absoption spectra of the groundwater colloids were collected, the 0.45-$\upmu$m polyethersulphone filters that contained the colloids had to be removed from the polyethylene capsule. This housing was removed using a Quick Release™ tubing cutter (Reed Pipe Tools and Vises); scalpels were then used to excise the filters. To avoid sample contamination, the excised filters (about 1.5 cm in thickness) were directly loaded onto Teflon XAS sample holders; each filter was tightly held between two sample holders using plastic screws. X-ray absorption spectra were collected at the Fe K-edge in fluorescence and transmission modes; the fluorescence signal was detected using a silicon drift detector (KETEK GmbH AXASM). For each sample three XAS scans were collected. At $\sim$200 eV below the Fe K-edge, an equidistant energy step of 10 eV was used; 0.5 eV equidistant energy steps were used 30 eV below the edge up until 40 eV above the edge. Beyond 40 eV relative to the edge, an equidistant k step of 0.03 Å with an integration time of 2 sec per point was used. In addition to the spectra of the colloidal samples, X-ray absorption spectra of Fe standard compounds were collected.

The HXMA beamline monochromator consisted of a Si(111) crystal adjusted to 1.5 mm x 3 mm. The monochromator was detuned by 50% to reduce higher harmonics and calibrated to the first inflection point of the K-edge (7112 eV) of an Fe reference foil. Soil samples were freeze dried, ground using ceramic mortar and pestle, and loaded onto Teflon XAS sample holders using Kapton tape. Spectra were collected in transmission and fluorescence modes at ambient temperature; fluorescence signals were measured using a 32-element Ge detector (Canderra); a Cu-6 filter and Soller slits were placed between the sample and the detector to reduce scattering and unwanted fluorescence from other elements. For each sample three scans were collected from 6912 eV to 7420.61 eV. The incident X-ray energy was scanned at 10 eV between 6912 and 7082 eV, 0.5 eV between 7082 and 7162 eV, and 0.05 k between 7162 and 7420.61 eV.

All spectra were processed and analyzed using Larch (version 0.9.68) Newville [30]. Iron pre-edge analysis was performed to estimate the amount of ferric and ferrous Fe in the soil and groundwater samples; linear combination fitting (LCF) was performed to determine the relative Fe mineral phases in the samples. A description of spectra processing and analyses is provided in Additional file 1.

### Mid-infrared spectroscopy

A Fourier transform infrared spectrometer (Bruker Corporation) with an attenuated total reflectance accessory was used to collect attenuated total reflectance Fourier transform infrared (ATR-FTIR) spectra from the GWCs and soil samples. The samples were freeze-dried, and the spectra were collected in the mid-infrared region (4000–400 cm$^{-1}$) with a 4 cm$^{-1}$ resolution. For each sample, an average spectrum was obtained from 256 scans using the OPUS Data Collection Software (Version 7.2) (Bruker Corporation).

## Results

### Chemistry of PHC-contaminated groundwater

In general, the chemistry of the PHC-contaminated groundwater slightly varied in space and time; however, no spatial gradient emerged between the amendment injection point and the farthest sampled monitoring well (Table 2). The groundwater had a neutral to slightly alkaline pH and was anoxic (the Eh strongly indicated the presence of Fe(III)-reducing conditions) (Table 2). Its dissolved Fe concentration was much lower than its total Fe concentration, suggesting that most of the Fe it contained was in the solid state (Table 2). Dissolved organic carbon and inorganic ions were present in appreciable amounts: sulfate, bicarbonate, and chloride were the dominant anions, whereas Ca$^{2+}$ and Mg$^{2+}$ were the dominant cations (Table 2).

### Redox state and coordination environment of Fe in GWCs and soils

**Fig. 1:**
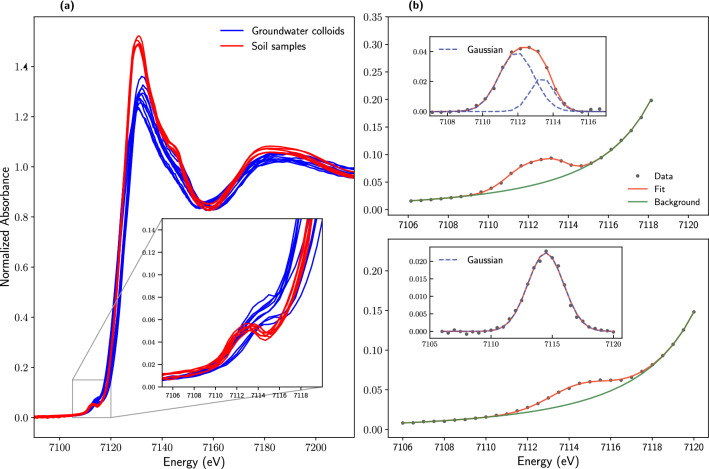
**a** Fe K-edge XANES spectra of groundwater colloids (blue lines) and soil samples taken from a three-meter soil core (red lines). The zoomed insert shows the position of the pre-edge features: of the two sample types, the pre-edge features of the soil samples are shifted to a lower energy. The three-meter soil core was taken $\sim$14.2 m from the injection point of the remedial solution, adjacent to one of the monitoring wells from which colloids were sampled. **b** The pre-edge features of Fe K-edge XANES spectra (black dots) of a soil sample (top) and a groundwater colloid (bottom). A linear + Lorentzian model (green line) was used to determine the background-corrected pre-edge intensities. The background-corrected pre-edges were either modeled with two Gaussian peaks [dashed blue lines in inserts] or modeled with one Gaussian peak. Red lines represent fitted pre-edge peaks. Centroids and integrated intensities estimated from fit models were used to estimate Fe(II) and Fe(III) percentages

The pre-edge features of the normalized Fe K-edge XANES was located at $\sim$7113 eV (Fig. 1a). The energy of the pre-edge feature of the GWCs appears to be higher than that of the soil samples. The background-corrected pre-edge peaks of a GWC and a soil sample and their Gaussian deconvolutions are shown in Fig. 1b; the fit parameters of the GWCs and soil samples are provided in Additional file 1: Tables S1 and S2, respectively. The pre-edge of all the GWCs was fitted with one component, but the pre-edge of each soil sample was fitted with either one or two components. The centroid and integrated area for the GWCs ranged between 7113.25$-$7114.47 eV, and 0.082$-$0.134 eV, respectively. For the soil samples, the centroid and integrated area ranged between 7112.23 and 7113.24 eV, and between 0.026 and 0.172 eV, respectively. To obtain a rough estimate of the redox state and coordination environment of Fe in the GWCs and the soils samples, two methods were used to estimate Fe$^{2+}/\Sigma$Fe and Fe$^{3+}/\Sigma$Fe ratios: the centroid-based calibration equation developed by Knipping et al., (21) and the intensity-centroid variogram developed by Wilke et al., (46). The results obtained from the two methods were consistent. According to the Knipping equation (Additional file 1: Equation 1), ${ ^{\text{VI}}\text{Fe}^{3+}}$ in both week-zero and week$-$22.5 GWCs ranged between 86% to 100% (Additional file 1: Table S1). The pre-edge parameters (total area and centroid) of all the GWCs plotted on the ${{ ^{\text{VI}}\text{Fe}^{2+}}}/{{^{\text{VI}}\text{Fe}^{3+}}}$ curve of the Wilke variogram, near the ${ ^{\text{VI}}\text{Fe}^{3+}}$ end member; this suggested that these samples contained mostly (>90%) octahedrally coordinated Fe$^{3+}$.

For the soil samples, $^{\text{IV}}\text{Fe}^{3+}$ was betweeen 13-71% and 9-43% for week-zero and week-22 samples, respectively (Additional file 1: Table S2). The pre-edge parameters for all the soils were located either on or near the ${ ^{\text{VI}}\text{Fe}^{2+}}/{ ^{\text{VI}}\text{Fe}^{3+}}$ curve. For the soils that were collected before the injection of the amendment solution (week zero), the pre-edge parameters of four of the samples were located about midway between the ${ ^{\text{VI}}\text{Fe}^{2+}}/{ ^{\text{VI}}\text{Fe}^{3+}}$ curve; that of five samples were located below the midway point (toward the ${ ^{\text{VI}}\text{Fe}^{2+}}$ end member), and that of 11 samples were located above the midway point (toward the ${ ^{\text{VI}}\text{Fe}^{3+}}$ end member). Therefore, for these samples, the octahedrally coordinated ${\text{Fe}^{3+}}$ was estimated to range between 15 to 70 %. For the soil samples that were collected 22.5 weeks after the continuous injection of the amendment solution, only one of the samples had their pre-edge parameters located at the 50:50 point of the ${ ^{\text{VI}}\text{Fe}^{2+}}/{ ^{\text{VI}}\text{Fe}^{3+}}$ curve; the other 19 samples had their parameters located at either the 60:40 point or below this point (toward the ${ ^{\text{VI}}\text{Fe}^{2+}}$ end member) with octahedrally coordinated ${\text{Fe}^{3+}}$ in these samples estimated to range between 10 and 50 percent. Thus, generally, the amount of octahedral ${\text{Fe}^{3+}}$ decreased with time.

**Table 2:** Groundwater chemistry of monitoring wells from which colloids were sampled

Parameter	EF–04$^1$		R1–12		R1–11		M17–08		M17–07	
	$^2$4.59 m		8.60 m		12.71 m		14.18 m		21.59 m	
	0 (weeks)	22.5 (weeks)	0 (weeks)	22.5 (weeks)	0 (weeks)	22.5 (weeks)	0 (weeks)	22.5 (weeks)	0 (weeks)	22.5 (weeks)
Temperature ($^{\circ }$C)	7.91	8.99	8.89	8.57	9.91	8.66	10.35	7.34	6.49	7.80
pH	7.69	7.23	7.80	7.23	7.86	7.27	7.83	7.23	7.84	7.48
EC (dS m$^{-1}$)	2.23	2.31	2.80	2.64	1.49	1.83	1.78	2.19	0.80	0.61
Eh (mV)	−29.5	−69.0	−50.9	−42.7	15.3	−18.3	15.7	−1.60	4.80	84.4
Organic C (mg L$^{-1}$)	45.00	25.00	0.10	14.00	0.50	12.00	24.00	100.0	40.00	315.00
Total Fe (mg L$^{-1}$)	687.0	70.90	43.20	61.40	49.20	308.0	377.00	391.0	215.0	632.0
Dissolved Fe (mg L$^{-1}$)	12.30	35.50	19.90	38.10	1.53	10.80	2.34	4.10	8.20	0.51
Total Mn (mg L$^{-1}$)	16.30	3.65	4.08	3.84	2.44	10.60	10.90	6.12	2.53	9.14
Dissolved Mn (mg L$^{-1}$)	2.43	3.14	3.17	3.21	1.41	3.13	1.52	1.61	2.05	0.69
Dissolved Si (mg L$^{-1}$)	13.00	10.60	11.10	13.00	9.64	12.30	9.71	10.90	10.90	4.89
Total P (mg L$^{-1}$)	6.39	1.45	0.68	1.45	0.84	0.18	0.38	11.80	4.00	13.60
${{\text{SO}^{2-}_{4}}}$ (mg L$^{-1}$)	330.0	1090	7.10	8.00	6.00	5.80	11.00	30.00	26.00	19.00
Sulfide (mg L$^{-1}$)	<0.002	<0.002	<0.002	<0.002	<0.002	<0.002	<0.002	<0.002	<0.002	<0.002
${\text{HCO}^{-}_{3}}$ (mg L$^{-1}$)	1040.0	477.0	845.0	960.0	637.0	975.0	922.0	1170.0	439.0	332.0
${\text{Cl}^{-}}$ (mg L$^{-1}$)	138.00	26.50	587.0	459.0	179.00	181.00	187.00	224.0	43.60	29.10
${\text{NO}^{-}_{3}}$ (mg L$^{-1}$)	<0.05	<0.05	<0.02	<0.05	<0.01	<0.05	<0.01	<0.05	0.07	<0.01
${\text{NO}^{-}_{2}}$ (mg L$^{-1}$)	<0.02	<0.02	<0.02	<0.02	<0.005	<0.02	<0.005	<0.02	0.06	<0.01
${\text{Ca}^{2+}}$ (mg L$^{-1}$)	374.00	278.0	322.0	322.0	219.00	267.00	267.00	358.0	116.0	63.70
${\text{Mg}^{2+}}$ (mg L$^{-1}$)	114.00	179.0	118.0	112.0	68.30	89.00	81.60	107.0	24.00	14.80
${\text{Na}^{+}}$ (mg L$^{-1}$)	113.00	84.40	87.30	106.0	31.60	11.00	14.00	20.00	36.10	37.50
${\text{K}^{+}}$ (mg L$^{-1}$)	2.40	5.00	5.30	7.90	4.80	5.50	2.70	4.00	2.50	2.90
Total Alkalinity (mg/L)	857	391	552	800	693	787	756	962	360	272
Ionic Balance (%)	8.59	1.58	5.39	0.15	1.37	2.14	0.26	2.84	2.35	4.36

$^1$ ID of monitoring well

$^2$ Distance between monitoring well and center of injection point

**Fig. 2:**
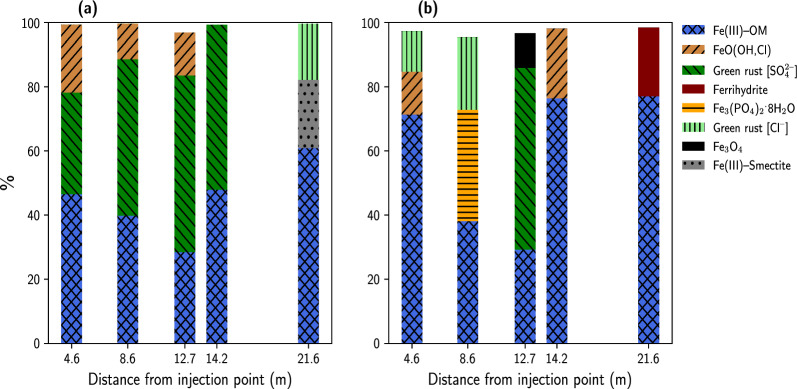
Relative proportions of Fe species present in groundwater colloids which were sampled at different distances from the point of injection of the remedial solution. **a** Groundwater colloids before injection of remedial solution (week-zero);** b** Groundwater colloids after 22.5 weeks of remedial solution injection. Species percentages were obtained from linear combination fitting (LCF) of Fe K-edge XANES. The blue bars, Fe(III)–OM, represent Fe(III)–organic matter coprecipitate

**Fig. 3:**
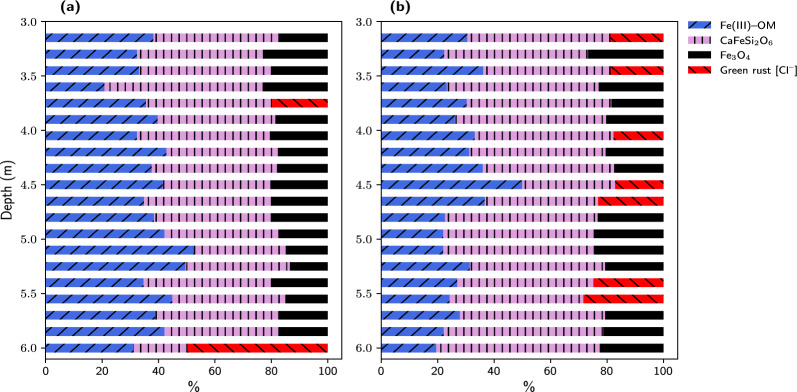
Relative proportions of Fe species present in soils which were sampled from a three-meter soil core. The three-meter soil core was taken $\sim$14.2 m from the injection point of the remedial solution, adjacent to one of the monitoring wells from which colloids were sampled. **a** Soil core taken before injection of the remedial solution (week-zero); **b** Soil core taken after 22.5 weeks of remedial solution injection. Species percentages were obtained from linear combination fitting (LCF) of Fe K-edge XANES. The blue bars, Fe(III)–OM, represent Fe(III)–organic matter coprecipitate

### Fe phases in groundwater colloids and soils

To determine the proportion of Fe phases in the GWCs and soil samples, linear combinations of Fe reference compounds were fitted to the normalized Fe K-edge XANES spectra of the samples (Additional file 1: Fig. S4 and S4). None of the samples analysed contained ferric ammonium citrate as a phase, suggesting that it was transformed to other phases (Figs. 2 and 3). All the week-zero GWCs had organic matter associated with Fe(III) [Fe(III)-OM] as a major phase, and all except one had sulfate green rust (GR [${{\text{SO}^{2-}_{4}}}$]) as a major phase. The colloids collected from the three monitoring wells closest to the injection gallery had akageneite [FeO(OH,Cl)] as a minor phase, but that from the farthest monitoring well had chloride green rust (GR [${\text{Cl}^{-}}$]) and Fe(III)-smectite as minor phases (Fig. 2a). Similar to the week-zero samples, the week$-$22.5 GWCs had Fe(III)-OM as a major phase; but, generally, the proportion of this phase was higher than in the week-zero GWCs (Fig. 2b). Sulfate green rust or vivianite was major phase in two week$-$22.5 GWCs. In addition, all week$-$22.5 GWCs contained either one or two of the following minor components: GR [${\text{Cl}^{-}}$], akageneite, magnetite (${\text{Fe}_{3}\text{O}_{4}}$), and ferrihydrite (Fig. 2b). For the soil samples, four major Fe phases were identified: Fe(III)-OM, magnetite, OM [${\text{Cl}^{-}}$], and ${\text{CaFeSi}_{2}\text{O}_{6}}$, a structural Fe(II) phase (Fig. 3a and b).

### ATR-FTIR spectroscopy

**Fig. 4:**
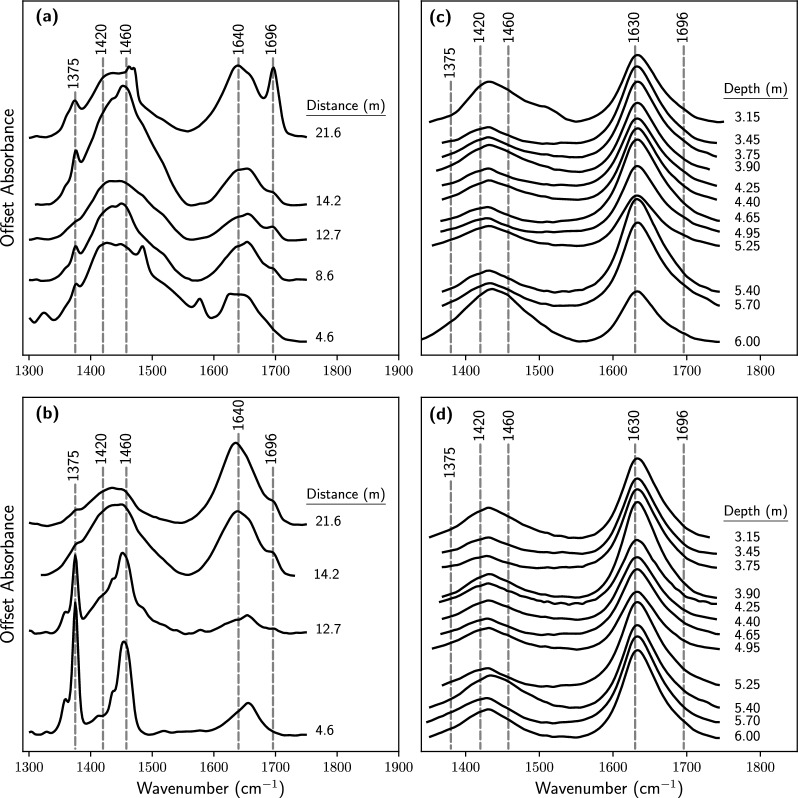
ATR-FTIR spectra of (**a**) groundwater colloids before injection of remedial solution (week-zero); **b** groundwater colloids after 22.5 weeks of remedial solution injection; **c** three-meter soil core taken before injection of the remedial solution (week-zero); **d** three-meter soil core taken after 22.5 weeks of remedial solution injection. The three-meter soil core was taken $\sim$14.2 m from the injection point of the remedial solution, adjacent to one of the monitoring wells from which colloids were sampled

The ATR-FTIR spectra of the GWCs and the soil samples were visually different (Fig. 4). The spectra of the former contained organic bands that were not visible in the spectra of the latter. The peak at 1696 cm$^{-1}$ was attributed to N–H bend in amine; the peak at 1640-1630 cm$^{-1}$ was due to O–H bend in adsorbed water. The peak at 1460 cm$^{-1}$ was attributed to C–H scissoring, and the peak at 1420 cm$^{-1}$ was due to C–O stretch. The peak at 1375 cm$^{-1}$ was attributed to COO–Fe(III) in either ${\text{Fe}^{3+}}$ adsorbed or coprecipitated with dissolved organic matter [8].

## Discussion

### Fe(III) transformation in hydrocarbon-contaminated groundwater

The results strongly suggest that ferric ammonium citrate [$({\text{NH}}_{4} )_{5} {\text{Fe}}({\text{C}}_{6} {\text{H}}_{4} {\text{O}}_{{\text{7}}} )_{2}$], the soluble compound that was injected into the PHC-contaminated groundwater, was transformed to solid Fe phases. In the structure of this compound, the central Fe atom is chelated by one hydroxyl group and two carboxyl groups from citrate; this prevents Fe(III) from hydrolysing, especially at circumneutral pH, and precipitating as Fe(III) (oxyhydr)oxides [27]. However, the relatively low concentration of soluble Fe in the groundwater (Table 2) suggested that the ferric component of $({\text{NH}}_{4} )_{5} {\text{Fe}}({\text{C}}_{6} {\text{H}}_{4} {\text{O}}_{7} )_{2}$ dissociated, hydrolyzed, and precipitated into solid Fe phases.

The composition and the redox state of the medium in which ${\text{Fe}^{3+}}$ precipitates determine the nature of solid Fe phases [14]. The parameters listed in Table 2 indicate that the hydrocarbon-contaminated groundwater had a neutral to slightly alkaline pH and was anoxic (Eh values were within the Fe(III) reducing range). In addition, the groundwater contained organic and inorganic ligands and dissolved cations. The Fe K-edge XANES and ATR-FTIR analyses showed that ${\text{Fe}^{3+}}$ reacted with some of these components to form solid phases. The presence of Fe(III)-OM in the GWCs (Fig. 2), and the presence of carboxyl–${\text{Fe}^{3+}}$ peaks in the ATR-FTIR spectra (Fig. 4) suggest that ${\text{Fe}^{3+}}$ coprecipitated and/or formed complexes with carboxyl groups. The association of organic matter with Fe(III) has been demonstrated in many laboratory experiments [6, 8, 29, 36] and has been found to occur in many surface and subsurface environments [22, 28, 45]. In soils, aquifers, and surface waters, aeration rapidly oxidizes ${\text{Fe}^{2+}}$ to ${\text{Fe}^{3+}}$; the latter hydrolyzes and either coprecipitates with DOM and/or precipitates then immedicably adsorbs DOM [29]. This process has been observed in acid sandy soils (pH 3.5$-$4.5) [31]; in peatlands where the rewetting of the surface oxic layer resulted in the formation of organo-Fe(III) coprecipitates [35]; and at the confluence of two streams, where precipitating Fe(III) oxides removed DOM from solution [28]. The results of this current study adds to the list of environments in which Fe(III)-OM associations can exist; they strongly suggest that Fe(III) can be associated with OM in an anoxic groundwater containing elevated levels of petroleum hydrocarbons.

GR [${{\text{SO}^{2-}_{4}}}$] and GR [${\text{Cl}^{-}}$] were major and minor phases, respectively, in the GWCs (Fig. 2). These layered double hydroxides contain alternating brucite-like layers in which part of the Fe(II) in the octahedral ${\text{Fe}(\text{OH})_{2}}$ layer is replaced by Fe(III). This substitution results in a positively charged layer which is countered by anions and structural water perched between the interlayers [15, 44]. In anoxic, circumneutral to alkaline environments (the PHC-contaminated groundwater being an example), green rusts can form via abiotic and biotic processes [44]. The former process involves the coprecipitation of Fe(III) (oxyhdr)oxides with ${\text{Fe}^{2+}}$, and the later process involves the bioreduction of Fe(III) (oxyhdr)oxides by dissimilatory Fe(III)-reducing bacteria [32, 37, 44]. The injection of the amendment solutions into the PHC-contaminated subsurface promoted the concurrent formation of Fe(III) (oxyhyr)oxides and Fe(III) reduction. The reduction of freshly precipitated Fe(III) (oxyhdr)oxides released ${\text{Fe}^{2+}}$, which, consequently, reacted with Fe(III) (oxyhdr)oxides and sulfate or chloride to form green rusts. Green rusts can also form when ${\text{Fe}^{2+}}$ and ${\text{Fe}(\text{OH})_{2}}$ are oxidized at pH 7–8 [41]. Because the Fe K-edge XAS spectra of the GWCs were not collected in an oxygen-free environment, and because the samples were not completely dry when the spectra were collected, the oxidation of ${\text{Fe}(\text{OH})_{2}}$ to GR [${{\text{SO}^{2-}_{4}}}$] and GR [${\text{Cl}^{-}}$] cannot be entirely ruled out.

The general chemical formula of GR is: ${[\mathrm{Fe(II)}_{1-\text{x}}\mathrm{Fe(III)}^{\text{x}}(\text{OH})_{2}]^{\text{x}+}\cdot [{ x/n} \text{A}^{\text{n}-}, \text{y} H_{2}\text{O}]^{\text{x}-}}$, where ${\text{A}^{\text{n}-}}$ represents the interlayer anions and *x* represents the molar fraction of Fe(III), which, stoichiometrically, ranges from 0.25 to 0.33 [1, 44]. However, the pre-edge analysis showed that the percentage of Fe(III) in all the GWCs was greater than 90, implying that the green rusts in these samples contained more than 33% Fe(III). The increase in the Fe(III) fraction of the green rusts in the GWCs is attributed to the partial oxidation of Fe(II) to Fe(III) during the collection of the XAS spectra.

Akageneite is an Fe(III)-oxyhydroxide polymorph which has chloride as part of its crystal structure: ${\text{FeO}_{0.833}(\text{OH})_{1.167}\text{Cl}_{0.167}}$[39]. Akageneite can precipitate when ${\text{Fe}^{3+}}$ hydrolyzes in chloride-rich solutions or when chloride-bearing green rust is oxidized [34]. The former process was probably responsible for the formation of Akageneite in the GWCs. But, as noted earlier, the oxidation of GR [${\text{Cl}^{-}}$] to akageneite cannot be completely ruled out as a transformation mechanism. The presence of vivianite in one of the colloidal samples suggests that some of the ${\text{Fe}^{2+}}$ released from Fe(III) bioreduction reacted with phosphate, which may have been released when sodium tripolyphosphate dissociated and/or when the phosphate-containing hydrochar particles were mineralized [11].

### Fe(III) transformation in hydrocarbon-contaminated subsoils

For the soil samples, the possibility of Fe(II) oxidation was minimized because they were freeze-dried (while frozen) before their XAS spectra were collected. Therefore, concerning the fraction of divalent and trivalent Fe in these samples, the results of the pre-edge and LCF analyses were generally consistent. Like the GWCs, all the soil samples contained Fe(III) associated with organic matter. The Fe(III)-OM coprecipitates that formed in the groundwater probably diffused into the soil matrix, where they were retained. In contrast to the GWCs, the average percentage of Fe(III)-OM in the two three-meter soil cores differed significantly: it decreased from 38.7% in the week-zero core to 28.7% in the week$-$22.5 core. The decrease in the fraction of Fe(III)-OM, together with the general decrease in ${ ^{\text{VI}}\text{Fe}(\text{III})}$ (as determined by the pre-edge analysis), suggests that the ferric component of this phase was being reduced by Fe(III)-reducing microorganisms. Many previous studies have characterized the nature of the Fe(III) phase in Fe(III)-OM coprecipitates and complexes. Using XRD and Mössbauer spectroscopy, Schwertmann et al., (2005) characterized an Fe(III)-OM coprecipitate that was synthesized using humic material that had been extracted from a Podzol. Their analysis showed that 4-line Fh was the main Fe(III) phase in the coprecipitate. Mikutta et al., (2008) found that 2-line ferrihydrite (Fh) precipitates in the presence of acid polysaccharides, and that the local coordination environment of Fh in the Fe(III)-OM coprecipitate was not significantly different from that of pure Fh. Similar results were recently reported by ThomasArrigo et al., (2019), who used shell fit analysis of Fe K-edge EXAFS to show that Fh in Fe(III)-OM and pure Fh shared a similar structure. Other researchers have reported that the XRD pattern of pure 2-line Fh and that of Fh in Fe(III)-OM are similar [7, 12]. Thus, it is very possible that in the GWCs and soil samples, Fh, or a poorly ordered Fe(III) oxyhydroxide, was associated with organic matter.

It has been reported that when Fe(III) and sulfate coexist in anoxic environments, microbial Fe(III) and sulfate reduction occur concurrently [5, 20]. The reduced species, ${\text{Fe}^{2+}}$ and sulfide, react to form mackinawite (FeS) [13, 33]. The extent of Fe(III) and sulfate reduction is controlled by solution pH; and, for Fe(III), the crystallinity of the (oxyhdr)oxides [13, 20]. At acidic to slightly alkaline pH, reduction of poorly crystalline Fe(III) (oxyhydr)oxides yields more energy than reduction of sulfate; thus, Fe(III) is reduced to a greater extent [13, 20, 33]. The results of this current study are generally consistent with those previously reported: sulfide and mackiwanite were below detection limits in the groundwater and subsoils, respectively. This strongly suggests that, in the slightly alkaline PHC-contaminated subsoils, Fe(III) reduction was highly favored over sulfate reduction.

The presence of magnetite and GR [${\text{Cl}^{-}}$] together with Fe(III)-OM further suggests that Fe(III) in the latter was being reduced by Fe(III)-reducing microorganisms. Magnetite usually forms in anaerobic environments in which bioreduction of Fe(III) occurs [14, 16, 17, 23, 48]. This was first reported by Lovley et al., (1987), who observed Fe(III)-reducing bacteria transform amorphous Fe(III) oxides to ultra-fine magnetite. Lovley and Phillips (1988) made similar observations after they had incubated, in an anaerobic medium, amorphous Fe(III) oxide or ferric citrate together with acetate and Fe(III)-reducing bacteria. After having studied the uncontaminated and PHC-contaminated sediments in the anoxic aquifer at Bemiji, MN, Zachara et al., (2004) reported that in the contaminated zone ferrihydrite-like phases were transformed to magnetite.

The presence of ${\text{CaFeSi}_{2}\text{O}_{6}}$ or ferrous calcium silicate is an indication that Fe(III) associated silicate minerals  were reduced in the PHC-contaminated soils. However, the amount of this phase in the week-zero and week$-$22.5 soils was similar, suggesting that most of it formed prior to the injection of the amendment solutions and ferric ammonium citrate. It is likely that following PHC contamination, microbes reduced Fe(III) structurally bound to silicate clays [40, 48].

### Iron geochemistry in PHC-contaminated soils and groundwater

In PHC-contaminated subsurface environments, years of natural attenuation results in the buildup of ${\text{Fe}^{2+}}$ at the expense of Fe(III) (oxyhydr)oxides [2, 24, 48]. Since ${\text{Fe}^{2+}}$ has the tendency to catalyze the transformation of amorphous ferric (oxyhdr)oxides, its abundance in these environments can cause freshly precipitated Fe(III) (oxyhydr)oxides to become less bioaccessible. Thus, the ferric amendment that was injected into the groundwater at the remedial site in Stony Plain was at risk of being transformed into more stable, less reducible forms.

It can be inferred from the results that Fe(II)-catalyzed transformation of Fe(III) (oxyhydr)oxides and its inhibition by organic matter probably occurred concurrently in the contaminated groundwater. The former process was likely responsible for the formation of chloride and sulfate green rust and akageneite. The high concentrations of chloride and sulfate in the groundwater (Table 2) may have favored the formation of these Fe phases over lepidocrocite and goethite. On the other hand, the coprecipitation of ferrihydrite with dissolved organic matter (and probably hydrochar particles) may have preserved the amorphous ferric iron by inhibiting its transformation by Fe(II). The formation of magnetite, especially in the soils, may have been due to two process: Fe(II)-catalyzed transformation of amorphous ferric oxides and microbial reduction of ferric phases including ferrihydrite bound to organic matter. However, given that its fraction was greater in the soils, where microbial activity was likely more pronounced, magnetite formation was likely mainly due to the microbial reduction of ferric phases.

## Conclusions

The current study sought to determine the transformation and fate of Fe(III) contributed by ferric ammonium citrate in an anoxic, PHC-contaminated subsurface environment. The colloids in the groundwater at this site were found to have contained ferric, ferrous, and mixed-valence solid Fe phases. Fe K-edge XANES analysis indicated that most of the injected Fe(III) coprecipitated with dissolved or particulate organic matter in the groundwater. The presence of GR [${\text{Cl}^{-}}$], GR [${{\text{SO}^{2-}_{4}}}$], and vivianite in the groundwater colloids suggested that the ferric phases served as electron acceptors during the microbial oxidation of petroleum hydrocarbons and other organic molecules. The subsoils from the site, like the groundwater colloids, contained Fe(III)-OM; this suggested that part of the Fe(III)-OM that coprecipitated in the groundwater was transported into the soil matrix. In contrast to the GWCs, the subsoils contained appreciable amounts of magnetite and ferrous calcium silicate, indicating that the extent of dissimilatory Fe(III) reduction was greater in this medium. Altogether, the results of this current study suggests that although ferric ammonium citrate did not persist in the PHC-contaminated subsurface, the Fe(III) it contained was transformed into solid phases that were bioaccessible to Fe(III)-reducing microbes.

## Supplementary Information

Additional file 1.

## Data Availability

The data supporting the conclusions of this article are included in the article and supporting information.
