# Supplementary material for: Transformation and fate of Fe(III) in petroleum-hydrocarbon-contaminated soil and groundwater
Source: Geochem Trans. 2025 Feb 7;26:1. doi: 10.1186/s12932-025-00097-z (PMC11806840; doi:10.1186/s12932-025-00097-z)
Supplement: Supplementary file 1 — Additional file 1. [file 12932_2025_97_MOESM1_ESM.pdf]

Transformation and fate of Fe(III) in petroleum-hydrocarbon-contaminated soil and groundwater

Essouassi Elikem[1*], David Bulmer[2], Kris Bradshaw[3], Ardalan Hayatifar[4], Matthew B.J. Lindsay[4], Steven Siciliano[2], Derek Peak[2]

[1*]Department of Chemistry, University of Saskatchewan, 170 Thorvaldson Building, Saskatoon, S7N 5E2, SK, Canada.
[2]Department of Soil Science, University of Saskatchewan, 51 Campus Drive, Saskatoon, S7N 5A8, SK, Canada.
[3]Federated Cooperatives Ltd., 401 22nd Street East, Saskatoon Saskatchewan, S7K 0H2, Canada
[4]Department of Geological Sciences, University of Saskatchewan, 114 Science Place, Saskatoon, S7N 5E2, SK, Canada.

*Corresponding author. E-mail: essouassi.elikem@usask.ca

Content	Page Number
Figure S1	5
Figure S2	6
Figure S3	7
Figure S4	8
Figure S5	9
Figure S6	10
Table S1	11
Table S2	11–14
Figure S5	14

Normalization of Fe K-edge XAS spectra

All data processing and modeling were performed using Larch (version 0.9.68) (Newville, 2013). All EXAFS spectra (standard and sample spectra) were

normalized in absorbance to remove the effects of sample preparation and thickness, concentration of absorber atom, and beamline configuration (e.g., detector sensitivity) (Kelly et al., 2008). Spectral features in the standards can be compared to those in the sample spectra only after these effects have been removed (Kelly et al., 2008). Briefly, individual scans of each spectrum were averaged to increase signal to noise ratio. A first-order polynomial was subtracted from the pre-edge whereas the post-edge was extrapolated to the adsorption-edge step of 1.0 by fitting either a first or a second order polynomial to post-edge region. Afterward, the pre-edge linear function was subtracted from the entire spectrum as background absorption; the background-subtracted data, at each energy, were divided by the edge step, which is the difference between the pre-edge and post-edge polynomials at E_0.

Pre-edge analysis of XANES spectra

The Fe K-edge XANES spectra of the soil and colloidal samples had peaks located about 10 eV below the main absorption edge. This pre-edge feature was produced by 1s→3d and/or 1s→4p metal electronic transitions, and it is very sensitive to the valence state of Fe (Dräger et al., 1988; Westre et al., 1997). Therefore, to determine the oxidation states of Fe in the soil and colloidal samples, the pre-edge feature of each sample spectrum was analyzed. First, the centroid (intensity-weighted average of pre-edge position) of the pre-edge peak or peaks was determined. Using part of the spectra between

7104 and 7118 eV, a linear plus Lorentzian function was used to determine the background, or the contribution of the main absorption edge to the pre-edge feature. Afterward, Gaussian functions were fitted to the background-subtracted pre-edge peak, and the fit parameters (amplitude and center) were used to calculate the centroid. All pre-edge peaks were modeled in Larch (version 0.9.68) (Newville, 2013). In this current study, $Fe^{2+}/\Sigma Fe$ and $Fe^{3+}/\Sigma Fe$ ratios were calculated using the centroid-based calibration equation developed Knipping et al., (2015) .

$$\frac{Fe^{2+}}{\Sigma Fe} = 1 - 0.5879 \times (centroid - 7111.9)^{1.2527} \tag{1}$$

Linear combination fitting of XANES spectra

The X-ray absorption near edge structure (XANES), the region of the XAS spectrum from ∼30 eV below the absorption edge to ∼50 eV above the absorption edge, is sensitive to the chemical species or oxidation states of the absorbing atom; and the chemical forms can be determined by regression of a linear combination of standard spectra to this region. Linear combination fitting (LCF) assumes that an XAS spectrum is a sum of the absorption from each chemical form in a sample (Kelly et al., 2008). Therefore, the percentages of Fe mineral phases in the GWCs and soil samples were determined by fitting a linear combination of Fe standard spectra to each experimental spectrum. All fitting were performed in Larch (version 0.9.68) (Newville, 2013). The LCF models were optimized by adjusting the fraction of stan-

dard spectra that contributed to the models. Energy shifts were not allowed in the fitting routines and the sum of fractions that contributed to the LCF model was not constrained to 1. To evaluate the accuracy of the LCF models, the XANES spectra of mixtures of Fe standards with known fractions were modeled; the fitted fractions of these mixtures were within $\pm10\%$ of the actual mole percentages. The XANES spectra of the Fe standards are shown in Additional file 1: Figure S3. Also, other factors were considered in the selection of best LCF model: (i) the redox state of Fe in the samples, as determined from the pre-edge peaks; (ii) the biogeochemical environment from which the samples were collected; (iii) the possible reactions of Fe in such an environment; (iv) number of standard spectra in LCF model (if two models had satisfactorily reconstructed a sample spectrum, the model with fewer standard spectra was selected).

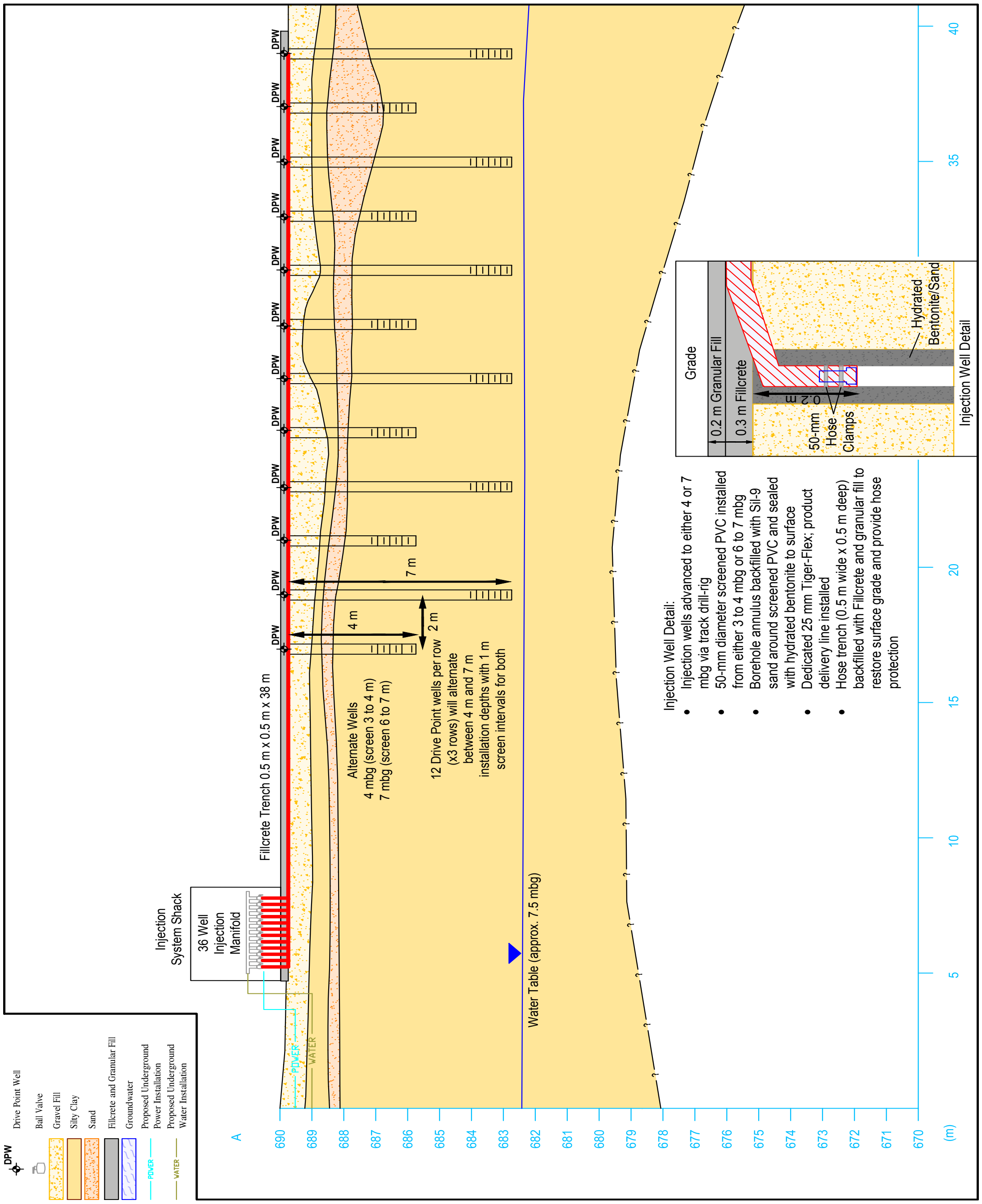

Figure S1. Schematic of injection gallery used to distribute amendment solutions at the contaminated site.

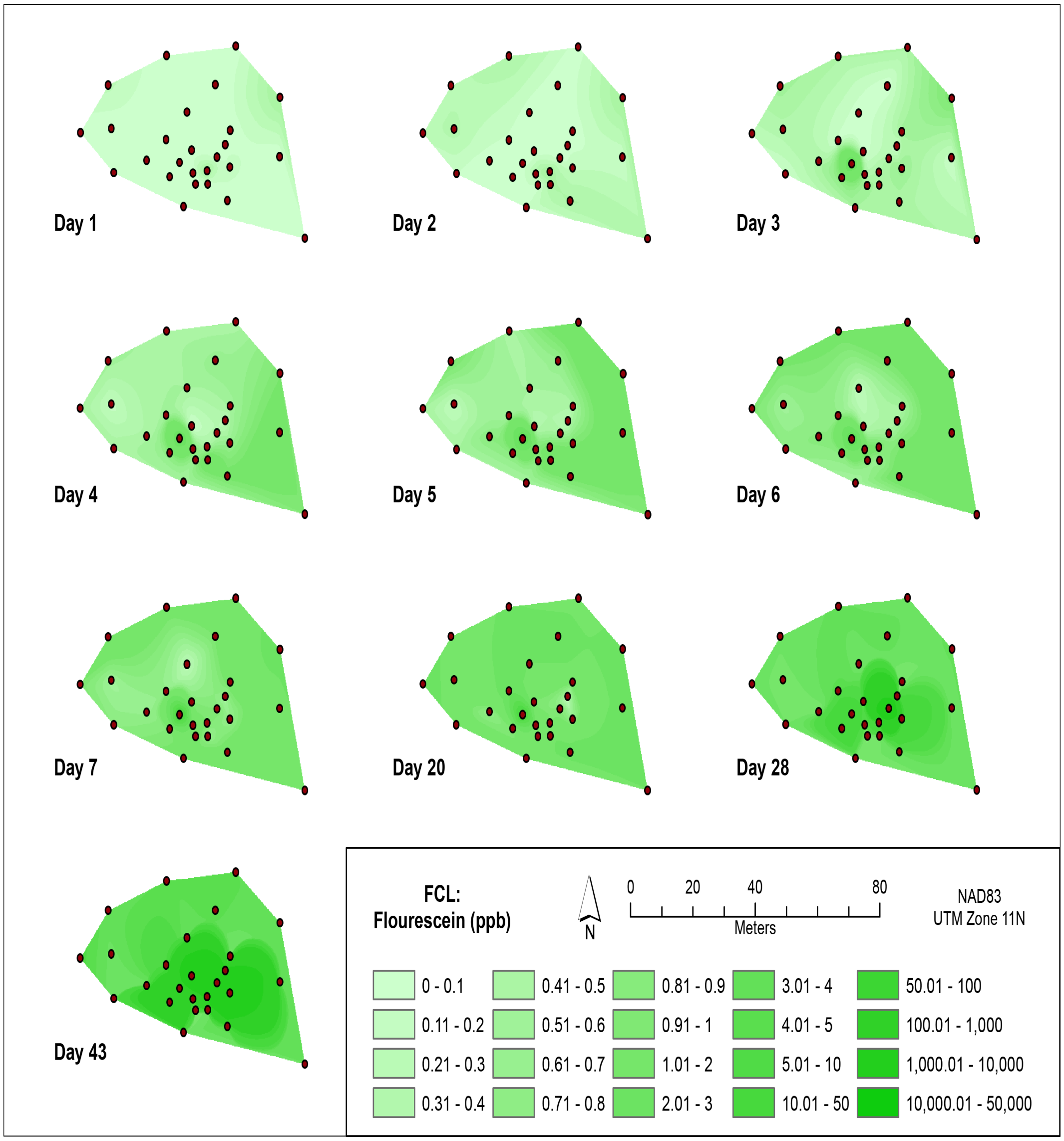

Figure S2. Spatial and temporal distribution of sodium flourescein at the PHC contaminated site. Red dots represent monitoring wells.

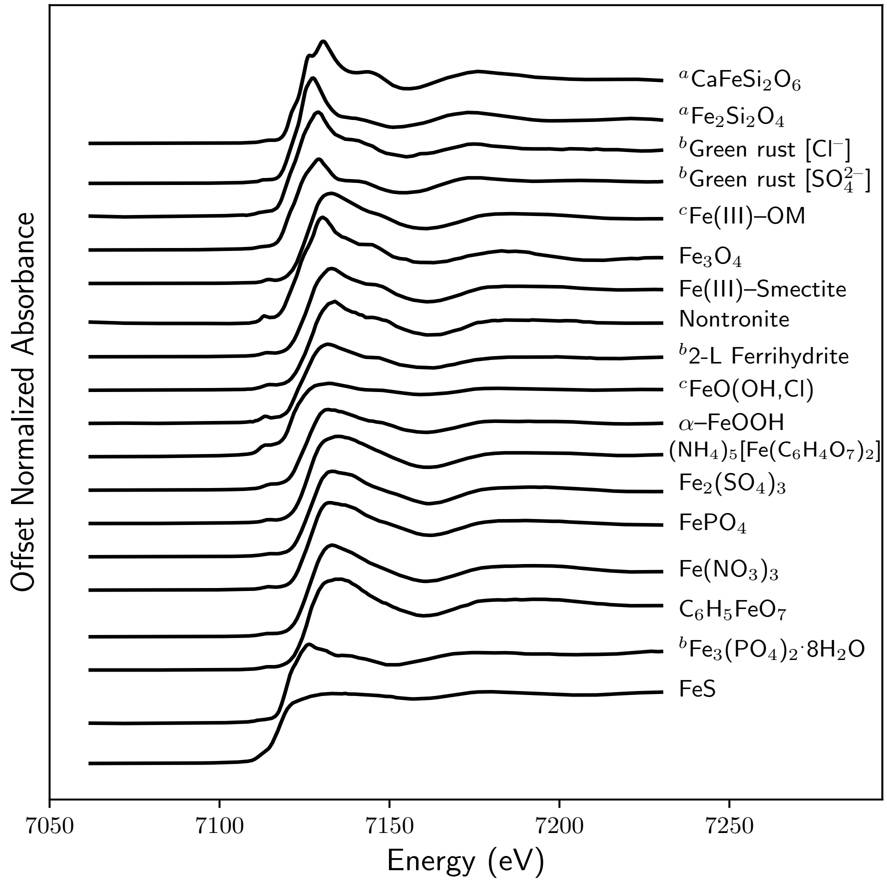

Figure S3. Fe K-edge XANES reference compounds used in linear combination fitting analysis. Spectra with superscript "a" were collected by Testemale and Sanchez-Valle, 2016a, 2016b; those with superscript "b" were collected by Hansel et al., 2003; those with superscript "c" were taken from http://ixs.iit.edu/database/data/Farrel_Lytle_data/RAW/Fe

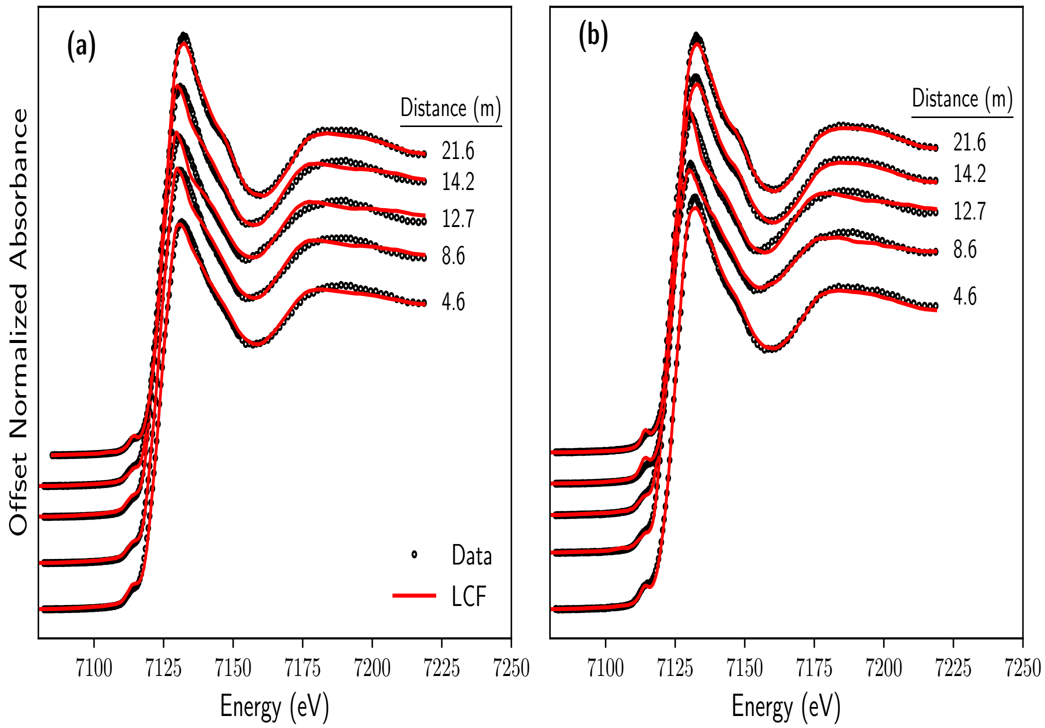

Figure S4. Fe K-edge XANES (small black circles) and linear combination fits (red lines) of (a) groundwater colloids before injection of remedial solution (week-zero); (b) groundwater colloids after 22.5 weeks of remedial solution injection.

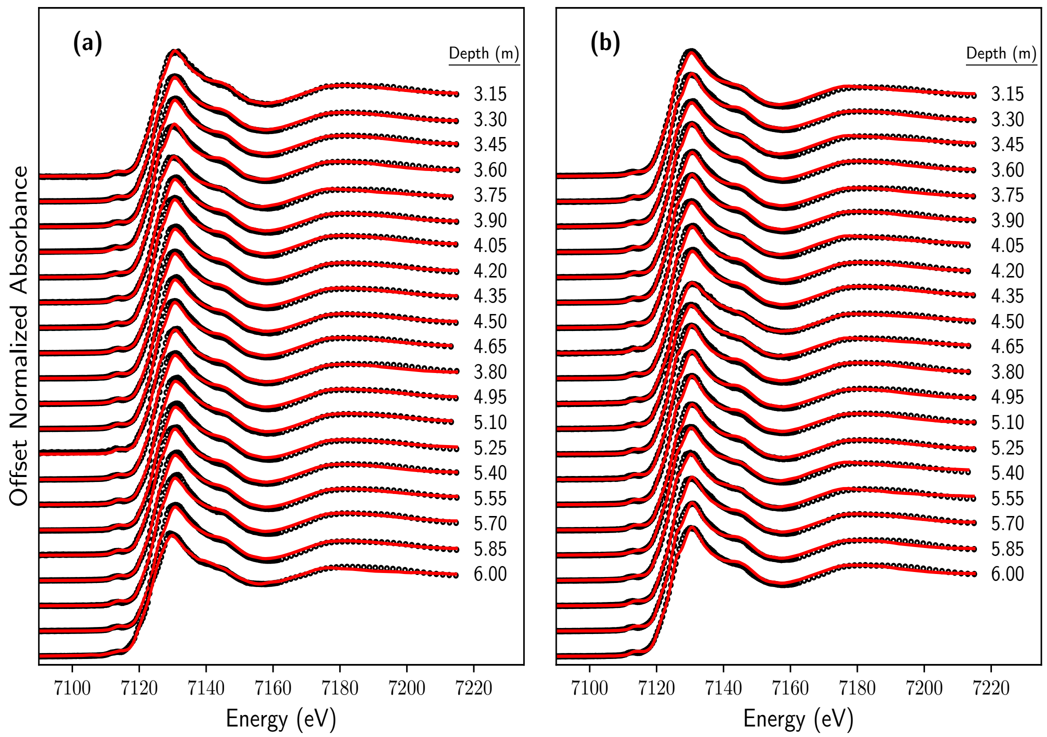

Figure S5. Fe K-edge XANES (small black circles) and linear combination fits (red lines) of (a) soil samples before injection of remedial solution (week-zero); (b) soil samples after 22.5 weeks of remedial solution injection. The three-meter soil cores were taken ∼14.2 m from the injection point of the remedial solution, adjacent to one of the monitoring wells from which colloids were sampled.

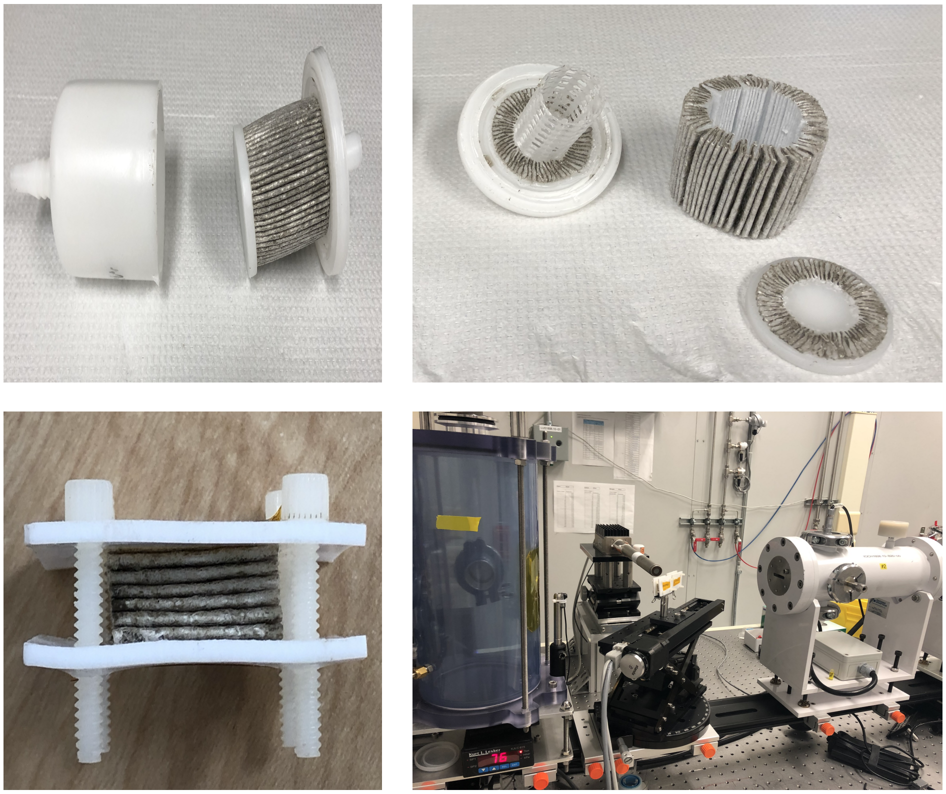

Figure S6. Top: 0.45 μm polyethersulphone filter containing groundwater colloids. Bottom left: filter excised and loaded into an EXAFS sample holder. Bottom right: Collection of extended X-ray absorption fine structure (EXAFS) spectra from two particulate samples at the IDEAS beamline of the Canadian Light Source (CLS).

Table S1. Pre-edge fit parameters and percentage of $^{VI}Fe^{3+}$ in groundwater colloids

Sample distance	Component parameters				Total area	Centroid	χ^2	$^{VI}Fe^{3+}$
	center	height	fwhm	area				
(m)	(eV)		(eV)	(eV)		(eV)		(%)
week-zero								
4.59	7113.36	0.026	3.935	0.109	0.109	7113.36	7.9x10^{-6}	94[1]
8.60	7113.38	0.021	3.887	0.086	0.086	7113.38	7.7x10^{-6}	96
12.71	7113.36	0.022	4.157	0.097	0.097	7113.36	7.1x10^{-6}	94
14.18	7113.26	0.022	3.759	0.088	0.088	7113.26	2.4x10^{-6}	86
21.59	7113.79	0.023	4.032	0.099	0.099	7113.79	7.5x10^{-6}	100
week-22.5								
4.59	7113.11	0.021	3.599	0.082	0.082	7113.11	5.1x10^{-6}	100
8.60	7113.37	0.028	4.422	0.134	0.134	7113.37	7.8x10^{-6}	95
12.71	7113.68	0.026	4.255	0.116	0.116	7113.68	2.0x10^{-6}	100
14.18	7113.25	0.023	3.742	0.092	0.092	7113.25	1.3x10^{-6}	100
21.59	7113.47	0.022	3.437	0.082	0.082	7113.47	9.9x10^{-6}	100

[1]Estimated using the centroid-based calibration equation (Knipping et al., 2015).

Table S2. Pre-edge fit parameters and percentage of $^{VI}Fe^{3+}$ and $^{VI}Fe^{2+}$ in soil samples

Sample distance	Component parameters				Total area	Centroid	χ^2	Fe_{tot}	$^{VI}Fe^{3+}$	$^{VI}Fe^{2+}$
	center	height	fwhm	area						
(m)	(eV)		(eV)	(eV)		(eV)		(%)	(%)	(%)
week-zero										
3.15	7112.48	0.026	2.077	0.057	0.079	7112.91	9.8x10^{-5}	5.3	50[1]	50
	7114.00	0.015	1.403	0.022						
3.30	7112.74	0.024	2.966	0.075	0.083	7112.85	1.06x10^{-5}	8.4	60	40

	7113.89	0.007	1.170	0.008						
3.45	7112.59	0.025	2.539	0.067	0.079	7112.81	$1.53x10^{-5}$	8.6	48	52
	7114.05	0.009	1.268	0.012						
3.60	7112.38	0.028	3.085	0.092	0.092	7112.38	$2.86x10^{-5}$	11.2	14	86
3.75	7112.34	0.026	3.060	0.085	0.085	7112.34	$3.00x10^{-5}$	8.2	13	87
3.90	7112.40	0.022	2.244	0.053	0.076	7112.83	$8.94x10^{-6}$	6.2	57	43
	7113.88	0.014	1.533	0.022						
4.05	7111.65	0.015	1.636	0.026	0.082	7112.76	$2.17x10^{-5}$	6.0	45	55
	7113.26	0.024	2.188	0.056						
4.20	7113.01	0.027	2.980	0.085	0.085	7113.01	$1.31x10^{-5}$	6.1	58	42
4.35	7112.22	0.020	2.291	0.050	0.078	7112.73	$6.31x10^{-6}$	7.1	44	56
	7113.64	0.015	1.742	0.028						
4.50	7112.96	0.026	3.148	0.088	0.088	7112.96	$2.99x10^{-5}$	6.0	58	42
4.65	7112.84	0.027	3.055	0.089	0.089	7112.84	$1.50x10^{-5}$	6.6	45	55
4.80	7112.97	0.029	2.535	0.078	0.078	7112.97	$3.70x10^{-4}$	5.5	61	39
4.95	7112.88	0.028	3.196	0.097	0.097	7112.88	$3.34x10^{-5}$	7.9	58	42
5.10	7113.24	0.028	3.001	0.089	0.089	7113.24	$3.69x10^{-5}$	6.8	71	29
5.25	7112.20	0.020	1.746	0.037	0.078	7113.07	$1.72x10^{-5}$	7.3	68	32
	7113.83	0.022	1.749	0.042						
5.40	7112.99	0.026	2.997	0.083	0.083	7112.99	$3.67x10^{-5}$	5.5	58	42
5.55	7112.55	0.023	2.403	0.059	0.083	7112.96	$8.17x10^{-6}$	6.6	59	41
	7113.99	0.013	1.632	0.023						
5.70	7112.11	0.023	1.953	0.047	0.076	7112.71	$2.55x10^{-5}$	6.3	48	52
	7113.69	0.019	1.436	0.029						
5.85	7112.90	0.027	3.057	0.086	0.086	7112.90	$1.88x10^{-5}$	9.4	51	49

6.00	7112.34	0.024	3.164	0.080	0.080	7112.34	7.18x10^{-5}	5.8	20	80
week-22.5										
3.15	7112.39	0.026	2.907	0.082	0.082	7112.39	1.77x10^{-5}	6.4	25	75
3.30	7113.36	0.014	1.459	0.022	0.075	7112.34	1.43x10^{-5}	6.6	22	78
	7111.91	0.023	2.165	0.053						
3.45	7112.45	0.027	2.940	0.085	0.085	7112.45	1.95x10^{-5}	9.6	20	80
3.60	7112.01	0.021	2.428	0.055	0.077	7112.40	4.35x10^{-6}	9.6	23	77
	7113.36	0.012	1.702	0.022						
3.75	7111.81	0.021	1.894	0.043	0.075	7112.46	6.40x10^{-6}	10.4	25	75
	7113.32	0.018	1.668	0.033						
3.90	7112.48	0.028	2.926	0.086	0.086	7112.48	1.34x10^{-5}	6.8	27	73
4.05	7112.42	0.028	2.883	0.085	0.085	7112.42	2.75x10^{-5}	7.7	22	78
4.20	7112.64	0.028	2.670	0.079	0.079	7112.64	2.09x10^{-4}	6.8	31	69
4.35	7112.51	0.026	2.875	0.081	0.089	7112.63	1.37x10^{-5}	7.4	43	57
	7113.84	0.006	1.218	0.008						
4.50	7111.91	0.021	1.802	0.041	0.094	7112.81	5.48x10^{-5}	7.4	42	58
	7113.50	0.023	2.125	0.053						
4.65	7112.56	0.029	2.921	0.091	0.091	7112.56	9.92x10^{-5}	6.1	26	74
4.80	7111.86	0.021	1.977	0.045	0.075	7112.46	1.28x10^{-5}	7.4	24	76
	7113.34	0.017	1.655	0.030						
4.95	7111.63	0.018	1.466	0.028	0.071	7112.59	6.10x10^{-5}	5.7	26	74
	7113.22	0.023	1.776	0.043						
5.10	7112.54	0.026	3.042	0.083	0.083	7112.54	1.62x10^{-5}	5.7	28	72
5.25	7111.96	0.020	1.759	0.038	0.074	7112.66	2.56x10^{-5}	6.6	40	60
	7113.39	0.019	1.784	0.036						

5.40	7113.22	0.013	1.595	0.022	0.077	7112.23	$1.16x10^{-5}$	9.3	12	88
	7111.84	0.022	2.344	0.055						
5.55	7112.23	0.026	3.046	0.083	0.083	7112.23	$2.93x10^{-5}$	8.7	9	91
5.70	7112.43	0.030	2.977	0.095	0.095	7112.43	$2.83x10^{-5}$	12.3	25	75
5.85	7112.36	0.027	2.829	0.080	0.080	7112.36	$3.39x10^{-5}$	7.3	21	79
6.00	7112.37	0.028	2.810	0.083	0.083	7112.37	$2.03x10^{-5}$	9.2	19	81

[1]Estimated using the centroid-based calibration equation (Knipping et al., 2015).

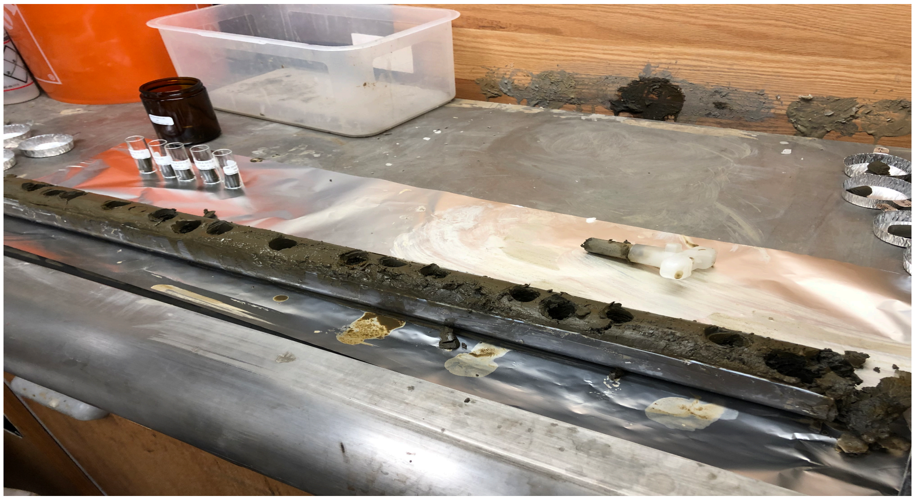

Figure S7. 0.75-m section of 3-m soil core being incrementally sampled using a Terra Core sampler. Five subsamples, weighing about 2 g, were collected at 15 cm intervals, and transferred into labelled glass vials.

References

Dräger, G., Frahm, R., Materlik, G., & Brümmer, O. (1988). On the multipole character of the X-ray transitions in the pre-edge structure of Fe K

absorption spectra. An experimental study. *physica status solidi (b)*, *146*, 287–294. https://doi.org/10.1002/PSSB.2221460130

Hansel, C. M., Benner, S. G., Neiss, J., Dohnalkova, A., Kukkadapu, R. K., & Fendorf, S. (2003). Secondary mineralization pathways induced by dissimilatory iron reduction of ferrihydrite under advective flow. *Geochimica et Cosmochimica Acta*, *67*, 2977–2992. https://doi.org/10.1016/S0016-7037(03)00276-X

Kelly, S. D., Hesterberg, D., & Ravel, B. (2008). Analysis of soils and minerals using x-ray absorption spectroscopy. In A. Ulery & L. R. Drees (Eds.), *Methods of soil analysis part 5—mineralogical methods* (pp. 387–463). John Wiley & Sons, Ltd. https://doi.org/10.2136/sssabookser5.5.c14

Knipping, J. L., Behrens, H., Wilke, M., Göttlicher, J., & Stabile, P. (2015). Effect of oxygen fugacity on the coordination and oxidation state of iron in alkali bearing silicate melts. *Chemical Geology*, *411*, 143–154. https://doi.org/https://doi.org/10.1016/j.chemgeo.2015.07.004

Newville, M. (2013). Larch: An analysis package for XAFS and related spectroscopies. *Journal of Physics: Conference Series*, *430*, 012007. https://doi.org/10.1088/1742-6596/430/1/012007

Testemale, D., & Sanchez-Valle, C. (2016a). Fe K edge xas transmission of natural hedenbergite $CaFeSi_2O_6$ at ambient conditions. *SSHADE/-FAME (OSUG Data Center) Dataset/Spectral Data.* https://www.sshade.eu/data/spectrum/SPECTRUM_DT_20170706_004

Testemale, D., & Sanchez-Valle, C. (2016b). Fe K edge xas transmission of synthetic fayalite Fe_2SiO_4 at ambient conditions. *SSHADE/FAME (OSUG Data Center) Dataset/ Spectral Data.* https://www.sshade.eu/data/spectrum/SPECTRUM_DT_20170706_003

Westre, T. E., Kennepohl, P., DeWitt, J. G., Hedman, B., Hodgson, K. O., & Solomon, E. I. (1997). A multiplet analysis of fe k-edge 1s $\rightarrow$ 3d pre-edge features of iron complexes. *Journal of the American Chemical Society*, *119*, 6297–6314. https://doi.org/10.1021/ja964352a
